# The effect of breathing exercises on fatigue in tuberculosis patients: a randomized controlled trial

**DOI:** 10.1590/1806-9282.20240888

**Published:** 2024-12-02

**Authors:** Sema Aytaç, Özlem Ovayolu, Sibel Dogru

**Affiliations:** 1 Gaziantep University, Faculty of Health Sciences, Department of Nursing – Gaziantep, Turkey.; 2 Gaziantep University, Faculty of Medicine, Department of Chest Diseases – Gaziantep, Turkey.

**Keywords:** Tuberculosis, Breathing exercises, Fatigue, Nursing

## Abstract

**OBJECTIVE:**

This study was conducted as a randomized controlled trial to investigate the effect of breathing exercises on fatigue in patients diagnosed with tuberculosis.

**METHODS:**

The tuberculosis patients included in the study were divided into two groups: intervention (26) and control (28) by a program established after the power analysis. After the researcher informed the patients in the intervention group about breathing exercises, including diaphragmatic and pursed lip breathing, they were taught, and the first exercise was practiced. Then, the patients in this group did breathing exercises once a day in the morning, 3 days a week, for a total of 4 weeks, with the researcher using the online interview method. The data were collected using a questionnaire and the Piper Fatigue Scale. The total score of the Piper Fatigue Scale ranges from 0 to 10, and the higher the score, the higher the fatigue level. The Piper Fatigue Scale was repeated at the end of the fourth week in both groups.

**RESULTS:**

Patients in the intervention group (88.5%) and control group (85.7%) reported that they were tired after the diagnosis of tuberculosis. The Piper Fatigue Scale total mean score of the intervention group was 8.29±1.19 before the intervention and dropped to 6.83±1.02 at the fourth week (p<0.05), whereas the Piper Fatigue Scale total mean score of the control group was 7.88±1.09 before the intervention and 7.93±1.02 at the fourth week (p>0.05).

**CONCLUSION:**

Breathing exercises done by tuberculosis patients were found to lower their levels of fatigue. Accordingly, it is recommended to benefit from breathing exercises, which are effective, inexpensive, and easy to apply in the management of fatigue.

**Clinical Trial Registration Number:**

The study was registered with clinicaltrial.gov NCT05202431.

## INTRODUCTION

Tuberculosis (TB) is a disease transmitted through the respiratory tract that is caused by the bacillus *Mycobacterium tuberculosis*. It mostly affects the lungs and is characterized by conditions such as cough, sputum, night sweats, shortness of breath, weakness, and getting tired quickly lasting for 2 weeks or longer^1^. Adherence to anti-tuberculosis treatment in this disease eliminates infectiousness and alleviates the symptoms of the patients generally until the disease is treated. However, it has been reported that fatigue, one of these symptoms, persists as a major problem^2^ and especially affects 88% of patients for whom treatment is ongoing and 47% of patients for whom treatment is completed^3^. Therefore, it is highly important to support patients with complementary approaches to cope with fatigue^4^. Today, methods such as pulmonary rehabilitation, including training, psychosocial support, and aerobic and strengthening breathing exercises, are utilized in these patients in addition to treatment^5^. The pursed lip breathing technique, one of the pulmonary rehabilitation practices, increases the activity of inspiratory and expiratory muscles, gas exchange, and tidal volume by providing a controlled expiration, thus helping to lower the respiratory rate. Diaphragmatic breathing exercise involves the use of the diaphragm muscle instead of auxiliary muscles; therefore, the respiratory load lessens, thus raising the ventilation level of the lungs and promoting breathing^6^. Breathing exercises, one of the methods used in the management of fatigue that emerges in chronic diseases, can help reduce symptoms specific to the disease by lessening the load on the respiratory muscles and allowing better oxygenation of the body with maximum ventilation^7,8^. Although it has been reported that these methods can be utilized to alleviate the fatigue that patients feel, the number of studies examining the effect of breathing exercises done by patients with pulmonary TB on fatigue is limited in the literature. Accordingly, this study was conducted as a randomized, controlled, and experimental trial to assess the effect of breathing exercises on fatigue levels in patients with pulmonary TB.

## METHODS

This randomized, controlled, and experimental study was conducted on patients with pulmonary TB registered at a tuberculosis dispensary in a province between June and September 2023.

### Population and sample of the study

The population of the study consisted of patients diagnosed with pulmonary TB registered in the tuberculosis dispensary, and the sample consisted of patients in the intervention (26) and control (28) groups according to the power analysis [α=0.05, the power of the test (1-β) was 0.80 and calculated as 21 in each group]^4,9^.

### Inclusion criteria

The patients who were literate, aged 18 years or older, had completed 15 days of pulmonary TB treatment, volunteered to participate in the study, had no communication problem, had no multiple drug resistance, and used a means of video communication were included in the study. The patients who failed to meet these criteria (n=60) were excluded from the study.

### Data collection

#### Questionnaire

The researchers created this form based on the literature^2,10^ and included questions about the socio-demographic and disease-related characteristics of the participants.

#### Piper fatigue scale

Piper et al. developed the scale, which consists of a total of 22 items and assesses the patients’ subjective perception of fatigue. The Turkish validity and reliability study of the scale was conducted by Can, and the reliability coefficient for the overall scale was reported as 0.94^11,12^. In this study, the Cronbach’s alpha value was found to be 0.987.

#### Implementation of the study

The Piper Fatigue Scale (PFS) was applied to the patients in a room available at the dispensary, and patients who got a score higher than “0” were assigned to the intervention and control groups using a simple random numbers table. After randomization, the researcher first informed the patients in the intervention group about the pursed lip and diaphragmatic breathing exercises through a face-to-face interview technique. Then, the breathing exercises were practically taught to each patient for approximately 30 min, and the first session was conducted in the presence of the researcher. The sessions lasted for 10–15 min and consisted of 10 pursed lip breathing exercises, relaxation (2 min), and 10 diaphragmatic breathing exercises. Except for the first session, the researcher reminded the patients of the sessions 3 days a week, once a day in the morning via online video call and on the other days by phone call, and made sure that the patients did the exercises. The researcher, who was trained and certified in breathing exercises, conducted the exercises for a total of 4 weeks. Nothing was applied to the patients in the control group. However, the researcher taught patients breathing exercises at the end of the study, and one session was applied.

### Ethical considerations

This study was conducted in accordance with the “Declaration of Helsinki,” and the necessary permissions were obtained from the ethics committee (2023/78), the provincial directorate of health, and the patients to collect the data.

### Statistical analysis

Data were assessed using the Statistical Package for Social Sciences-SPSS 25.0 packaged software. Frequency, percentage, mean, and standard deviation were used as descriptive statistics, and the Shapiro-Wilk test was used to determine the compatibility of continuous variables for normal distribution. Student’s t-test, chi-square, Mann-Whitney U, and Kruskal-Wallis tests were used to examine the differences between categorical variables. The statistical significance level was accepted as p<0.05.

## RESULTS

### Analysis of some characteristics of the patients

It was found that the mean ages of the patients in the intervention and control groups were 40.03±16.28 and 45.07±14.88 years, respectively. A total of 34.6% of the patients in the intervention group were female, and 38.5% had another family member with TB. Additionally, 39.3% of the patients in the control group were female, 42.9% graduated from primary school, and 10.7% had another family member with TB (Table 1).

**Table 1:** Comparison of some characteristics of patients.

Characteristics	Intervention group	Control group	p
	n (%)	n (%)	
Age (mean±SD)	40.03±16.28	45.07±14.88	
Gender			0.723
Female	9 (34.6)	11 (39.3)	
Male	17 (65.4)	17 (60.7)	
Educational level			0.197
Literate	5 (19.2)	10 (35.7)	
Primary school	10 (38.5)	12 (42.9)	
High school	11 (42.3)	6 (21.4)	
Profession			0.169
Worker	11 (42.3)	6 (21.4)	
Retired	3 (11.5)	5 (17.9)	
Unemployed	12 (46.2)	17 (60.7)	
Marital status			0.914
Married	18 (69.2)	19 (67.9)	
Single	8 (30.8)	9 (32.1)	
Cohabitation			0.549
Alone	4 (15.4)	5 (17.9)	
With family	22 (84.6)	23 (82.1)	
Income level			0.590
Good	2 (7.7)	2 (7.1)	
Moderate	11 (42.3)	8 (28.6)	
Poor	13 (50.0)	18 (64.3)	
Smoking			0.467
Yes	12 (46.2)	11 (39.3)	
No	9 (34.6)	14 (50.0)	
Quit smoking	5 (19.2)	3 (10.7)	
Comorbidity			0.310
Yes	6 (23.1)	10 (35.7)	
No	20 (76.9)	18 (64.3)	
Another family member with TB			**0.017**
Yes	10 (38.5)	3 (10.7)	
No	16 (61.5)	25 (89.3)	
Family member with TB (n:13)	n=10	n=3	0.077
Spouse	–	2 (66.7)	
Mother	2 (20.0)	–	
Father	4 (40.0)	–	
Child	2 (20.0)	–	
Sibling	2 (20.0)	1 (33.3)	
Total	26 (100)	28 (100)	

SD: standard deviation; TB: tuberculosis. Bold indicates p<0.05.

### Analysis of some circumstances related to tuberculosis in patients

In the intervention group, 38.5% of the patients were treated for “0–2 months,” 92.3% of them regularly used their medications, and 53.8% of them had difficulties in taking their TB medication. In the control group, 46.4% of the patients were treated for “0–2 months,” 96.4% of them regularly used their medications, and 64.3% of them had difficulties in taking their TB medication (Table 2).

**Table 2:** Comparison of some circumstances related to tuberculosis in patients.

Characteristics	Intervention group	Control group	p
Length of treatment			0.554
0–2 months	10 (38.5)	13 (46.4)	
3–6 months	16 (61.5)	15 (53.6)	
Receiving training in TB			0.872
Yes	19 (73.1)	21 (75.0)	
No	7 (26.9)	7 (25.0)	
Regular use of TB medication			
Yes	24 (92.3)	27 (96.4)	0.604
No	2 (7.7)	1 (3.6)	
Having difficulty in taking TB medication			0.435
Yes	14 (53.8)	18 (64.3)	
No	12 (46.2)	10 (35.7)	
How to assess the disease			0.130
I think I will never recover	1 (3.8)	6 (21.4)	
I think I can recover	17 (65.4)	12 (42.9)	
I do not know	8 (30.8)	10 (35.7)	
Suffering side effects due to medication			0.104
Yes	12 (46.2)	7 (25.0)	
No	14 (53.8)	21 (75.0)	
Side effects suffered			**0.037**
Itching	7 (58.3)	2 (28.6)	
Loss of appetite	3 (25.0)	1 (14.3)	
Dizziness	2 (16.7)	–	
Abdominal pain	–	4 (57.1)	
Total	26 (100)	28 (100)	

TB: tuberculosis. Bold indicates p<0.05.

### Analysis of the patients’ fatigue- related characteristics

A total of 88.5% of the patients in the intervention group and 85.7% of the patients in the control group complained of fatigue. Also, the PFS mean scores of the patients in the intervention group at baseline and at the end of the first month were 8.29±1.19 and 6.83±1.02, respectively. The PFS mean score declined after the breathing exercises, and the difference between the two measurements was statistically significant (p<0.05). The PFS mean scores of the patients in the control group at baseline and at the end of the first month were 7.88±1.09 and 7.93±1.02, respectively. The PFS mean score did not change, and the difference between the two measurements was not statistically significant (p>0.05) (Table 3).

**Table 3:** Comparison of patients’ characteristics related to fatigue.

Characteristics	Intervention group	Control group	p
PFS mean scores of the patients during the follow-up period	PFS Mean±SD	PFS Mean±SD	
Baseline PFS score	8.29±1.19	7.88±1.09	0.105^z^
Final PFS score	6.83±1.02	7.93±1.02	**0.001^z^**
p (intra-group)	**0.000**	0.164	
Fatigue after the diagnosis of TB			0.543
Yes	23 (88.5)	24 (85.7)	
Occasionally	3 (11.5)	4 (14.3)	
The time of day when fatigue is most common			0.161
Morning	10 (38.5)	18 (64.3)	
Noon	1 (3.8)	2 (7.1)	
Evening	4 (15.4)	3 (10.7)	
Constant	11 (42.3)	5 (17.9)	
Difficulty in taking medication when tired			0.439
Yes	17 (65.4)	21 (75.0)	
No	9 (34.6)	7 (25.0)	
Factors that increase fatigue			0.143
Moving	11 (42.3)	18 (64.3)	
Working	7 (26.9)	7 (25.0)	
So tired even without doing anything	8 (30.8)	3 (10.7)	
Factors that alleviate fatigue			0.169
Resting	19 (73.1)	25 (89.3)	
Nothing alleviates	7 (26.9)	3 (10.7)	
Effect of fatigue on quality of life			**0.026**
Too much	14 (53.8)	9 (32.1)	
Moderate	8 (30.8)	18 (64.3)	
Less	3 (11.5)	–	
No effect	1 (3.8)	1 (3.6)	
Effect of fatigue on nutrition			**0.041**
Too much	11 (42.3)	10 (35.7)	
Moderate	8 (30.8)	16 (57.1)	
Less	5 (19.2)	–	
No effect	2 (7.7)	2 (7.1)	
Effect of fatigue on sleep			0.115
Too much	7 (26.9)	12 (42.9)	
Moderate	8 (30.8)	12 (42.9)	
Less	5 (19.2)	3 (10.7)	
No effect	6 (23.1)	1 (3.6)	
Effect of fatigue on the performance of roles			0.494
Yes	20 (76.9)	24 (85.7)	
No	6 (23.1)	4 (14.3)	
Total	26 (100)	28 (100)	

TB: tuberculosis; PFS: Piper Fatigue Scale; SD: standard deviation. Bold indicates p<0.05. ^z^Mann-Whitney U test.

## DISCUSSION

Clinical symptoms of pulmonary TB develop slowly and are usually non-specific^13^. Patients who are under treatment and even those who have been cured have been reported to suffer from fatigue, one of the most prevalent symptoms of pulmonary TB^3,4^. A qualitative study on this subject reported that the patient stated, “*After I took the drugs, I got tired and could not work properly, and this caused financial loss. That is why I stopped taking the medication*”^14^. This shows that fatigue is an important problem for TB patients. However, it has been known that the number of studies on the effect of breathing exercises, one of the methods used to alleviate fatigue, in patients with pulmonary TB is very limited. Therefore, this study examined the effect of breathing exercises on fatigue in patients with pulmonary TB.

In the literature, it has been reported that fatigue is a prevalent symptom in patients with pulmonary TB and affects approximately 88% of patients^2^. The disease reduces lung capacity and induces obstructive airway disorders, leading to inadequacy in gas exchange and difficulty breathing, and thus fatigue may appear in patients^4^.

It has been emphasized that it complicates medication adherence, especially when patients suffer from fatigue along with other side effects while using antituberculosis drugs^15^. In their study, Wahyudı et al. reported that deep breathing exercises had a significant effect on lowering the respiratory rate in patients with pulmonary TB^10^.

The study by Namuwali et al. indicated that TB patients tended to suffer from emotional disturbance due to the disease, and deep breathing exercises applied for 3 weeks were effective in controlling the emotions of TB patients^16^. The study by Nguantad et al., which examined the effect of a 6-week integrated rehabilitation program on fatigue, revealed that the integrated rehabilitation program significantly lowered the fatigue level of patients within 3–6 weeks^4^. Another study reported that the use of spiritually emotional breathing techniques lowered interleukin-2, cortisol levels, and immunoglobulin G in patients, and they can be applied as a complementary treatment to enhance the quality of life and control symptoms^17^. This study also revealed that breathing exercises applied by the intervention group significantly decreased the PFS mean score, whereas the level of fatigue did not change in the control group. Furthermore, 88.5% of the patients in the intervention group and 85.7% of the patients in the control group suffered from fatigue after the diagnosis of TB, and 65.4% of the patients in the intervention group and 75.0% of the patients in the control group had difficulties in taking medication when tired. These results suggested that fatigue should be regularly assessed and measures should be taken for effective treatment management and prevention of infectiousness in patients diagnosed with pulmonary TB.

In the literature, it has been reported that the quality of life of TB patients is significantly affected, and this effect persists after treatment^18^. A study conducted on this topic reported that 70% of TB patients had poor sleep quality^19^. It was also indicated that sleep quality interacts with nutrition and immunity^20,21^. This study also reported that fatigue in TB patients drastically affected their quality of life, nutrition, sleep, and performance in their roles. It clearly shows that the fatigue that appears with the disease can negatively impact these factors, particularly due to side effects related to medication use.

During the 6–9 months of TB treatment, patients may have difficulty in medication adherence due to these factors, and therefore treatment may be interrupted. Accordingly, it is recommended to make use of breathing exercises in the management of symptoms such as fatigue that TB patients may suffer from during and after treatment.

## CONCLUSION AND RECOMMENDATIONS

This study, which examined the effect of breathing exercises that patients with pulmonary TB did for 4 weeks on fatigue, revealed that the breathing exercises applied in the intervention group decreased the total PFS mean scores of the patients, whereas the mean PFS scores of the patients in the control group did not change. Furthermore, fatigue was identified as a major problem for the patients, and 88.5% of the patients in the intervention group and 85.7% of the patients in the control group suffered from fatigue and had difficulty in taking TB medication when they were tired. Furthermore, fatigue negatively affected quality of life, nutrition, sleep, and performance of roles in a great majority of patients. Based on these results, it may be recommended to regularly assess the fatigue experienced by patients with pulmonary TB from the time of diagnosis, consider the areas that fatigue may affect, and use breathing exercises, which are easy to apply, inexpensive, effective, and tolerable methods, in the management of fatigue.

### Limitations

The most important limitation of this study is that “fatigue” was assessed only using a scale, and the results are specific to the region where the study was conducted.

## References

[1] Saglik Bakanlıgı TC (2019). Tüberküloz tani ve tedavi rehberi.

[2] Thedthong W, Puwarawuttipanich W, Saneha C, Rongrunguang Y (2021). Factors predicting fatigue in pulmonary tuberculosis patients receiving anti-tuberculosis drugs. Siriraj Med J.

[3] Lin Y, Liu Y, Zhang G, Cai Q, Hu W, Xiao L (2021). Is it feasible to conduct post-tuberculosis assessments at the end of tuberculosis treatment under routine programmatic conditions in China?. Trop Med Infect Dis.

[4] Nguantad S, Puwarawuttipanit W, Lekdamrongkul P, Rongrungruang Y (2023). Effects of an integrated rehabilitation program on fatigue in patients with pulmonary tuberculosis. Siriraj Med J.

[5] Kara D, Ertürk A, Gürsel A, Köktürk F, Yıldız H, Akansel N (2013). The effect of pursed lip and diaphragmatic breathing exercises applied to chronic obstructive pulmonary patients on dyspnea severity and pulmonary function tests.. Anat J Nurs Health Sci.

[6] Akıncı AÇ, Pınar R (2012). Kronik obstrüktif akciğer hastaliği olan hastalarda dispne rehabilitasyonu.. Cumhuriyet Hemşirelik Dergisi.

[7] Çakar F, Simşek H, Sever A (2018). The effect of diaphragmatic breathing exercise on some mental and physical health levels in young people.. Tr J Nat Sci..

[8] Kıskaç N, Katran HB, Manav G, Türkoğlu BE, Yokarıbaş E (2023). Hastalarda yorgunluk semptomunun yönetiminde non-farmakolojik yöntemlerin etkisi-sistematik derleme. J Acad Res Nurs.

[9] Jang KS, Oh JE, Jeon GS (2022). Effects of simulated laughter therapy using a breathing exercise: a study on hospitalized pulmonary tuberculosis patients. Int J Environ Res Public Health.

[10] Wahyudı DA, Xanda AN, Sukesı N, Puspita L, Wardani PK, Yurlina E (2021). Active cycle of breathing to respiratory rate in patients with lung tuberculosis. Int J Pharm Res.

[11] Piper BF, Dibble SL, Dodd MJ, Weiss MC, Slaughter RE, Paul SM (1998). The revised Piper Fatigue Scale: psychometric evaluation in women with breast cancer. Oncol Nurs Forum.

[12] Can G, Durna Z, Aydiner A (2004). Assessment of fatigue in and care needs of Turkish women with breast cancer. Cancer Nurs.

[13] Luies L, Preez I (2020). The echo of pulmonary tuberculosis: mechanisms of clinical symptoms and other disease-induced systemic complications.. Clin Microbiol Rev..

[14] Bhattacharya T, Ray S, Biswas P, Das D (2018). Barriers to treatment adherence of tuberculosis patients: a qualitative study in West Bengal, India. Int J Med Sci Public Health.

[15] Amalba A, Bugri AA (2021). Assessing the prevalence and effect of adverse drug reactions among patients receiving first line anti-tubercular medicines in the Tamale Teaching Hospital, Ghana. Pan Afr Med J.

[16] Namuwali D, Mendrofa F, Dwidiyanti M (2016). Deep breathing relaxation techniques improve emotional control on tuberculosis patients.. Int J Public Health Sci.

[17] Kusnanto K, Haryanto J, Sukartini T, Ulfiana E, Putra MM, Ners J (2018). The effectiveness of spiritual emotional breathing towards respiratory function and immune response of tuberculosis patients. J Ners.

[18] Guo N, Marra F, Marra CA (2009). Measuring health-related quality of life in tuberculosis: a systematic review. Health Qual Life Outcomes.

[19] Liu X, Lan H, Bai X, Li Q, Wen Y, Feng M (2023). Sleep quality and its associated factors among patients with tuberculosis: a cross-sectional study. Front Public Health.

[20] Besedovsky L, Lange T, Haack M (2019). The sleep-immune crosstalk in health and disease. Physiol Rev.

[21] Doherty R, Madigan S, Warrington G, Ellis J (2019). Sleep and nutrition interactions: implications for athletes. Nutrients.

