# Erratum

**DOI:** 10.1590/1806-9282.20240888ERRATUM

**Published:** 2025-01-20

In the manuscript "The effect of breathing exercises on fatigue in tuberculosis patients: a randomized controlled trial", https://doi.org/10.1590/1806-9282.20240888, published in the Rev Assoc Med Bras. 2024;70(12):e20240888, on page 3:

**Where it reads:**

**Table 1:** Comparison of some characteristics of patients.

Characteristics		Intervention group	Control group	p
		n (%)	n (%)	
Age (mean±SD)		40.03±16.28	45.07±14.88	
Gender				0.723
	Female	9 (34.6)	11 (39.3)	
	Male	17 (65.4)	17 (60.7)	
Educational level				0.197
	Literate	5 (19.2)	10 (35.7)	
	Primary school	10 (38.5)	12 (42.9)	
	High school	11 (42.3)	6 (21.4)	
Profession				0.169
	Worker	11 (42.3)	6 (21.4)	
	Retired	3 (11.5)	5 (17.9)	
	Unemployed	12 (46.2)	17 (60.7)	
Marital status				0.914
	Married	18 (69.2)	19 (67.9)	
	Single	8 (30.8)	9 (32.1)	
Cohabitation				0.549
	Alone	4 (15.4)	5 (17.9)	
	With family	22 (84.6)	23 (82.1)	
Income level				0.590
	Good	2 (7.7)	2 (7.1)	
	Moderate	11 (42.3)	8 (28.6)	
	Poor	13 (50.0)	18 (64.3)	
Smoking				0.467
	Yes	12 (46.2)	11 (39.3)	
	No	9 (34.6)	14 (50.0)	
	Quit smoking	5 (19.2)	3 (10.7)	
Comorbidity				0.310
	Yes	6 (23.1)	10 (35.7)	
	No	20 (76.9)	18 (64.3)	
Another family member with TB				**0.017**
	Yes	10 (38.5)	3 (10.7)	
	No	16 (61.5)	25 (89.3)	
Family member with TB (n:13)		n=10	n=3	0.077
	Spouse	–	2 (66.7)	
	Mother	2 (20.0)	–	
	Father	4 (40.0)	–	
	Child	2 (20.0)	–	
	Sibling	2 (20.0)	1 (33.3)	
	Total	26 (100)	28 (100)	

SD: standard deviation; TB: tuberculosis. Bold indicates p<0.05.

**It should read:**

**Table 1:** Comparison of some characteristics of patients.

Characteristics		Intervention group	Control group	p
		n (%)	n (%)	
Age (mean±SD)		40.03±16.28	45.07±14.88	0.302
Gender				0.723
	Female	9 (34.6)	11 (39.3)	
	Male	17 (65.4)	17 (60.7)	
Educational level				0.197
	Literate	5 (19.2)	10 (35.7)	
	Primary school	10 (38.5)	12 (42.9)	
	High school	11 (42.3)	6 (21.4)	
Profession				0.169
	Worker	11 (42.3)	6 (21.4)	
	Retired	3 (11.5)	5 (17.9)	
	Unemployed	12 (46.2)	17 (60.7)	
Marital status				0.914
	Married	18 (69.2)	19 (67.9)	
	Single	8 (30.8)	9 (32.1)	
Cohabitation				0.549
	Alone	4 (15.4)	5 (17.9)	
	With family	22 (84.6)	23 (82.1)	
Income level				0.590
	Good	2 (7.7)	2 (7.1)	
	Moderate	11 (42.3)	8 (28.6)	
	Poor	13 (50.0)	18 (64.3)	
Smoking				0.467
	Yes	12 (46.2)	11 (39.3)	
	No	9 (34.6)	14 (50.0)	
	Quit smoking	5 (19.2)	3 (10.7)	
Comorbidity				0.310
	Yes	6 (23.1)	10 (35.7)	
	No	20 (76.9)	18 (64.3)	
Another family member with TB				**0.017**
	Yes	10 (38.5)	3 (10.7)	
	No	16 (61.5)	25 (89.3)	
Family member with TB (n:13)		n=10	n=3	0.077
	Spouse	–	2 (66.7)	
	Mother	2 (20.0)	–	
	Father	4 (40.0)	–	
	Child	2 (20.0)	–	
	Sibling	2 (20.0)	1 (33.3)	
	Total	26 (100)	28 (100)	

SD: standard deviation; TB: tuberculosis. Bold indicates p<0.05

